# Comparison of 1-week and 2-week recall periods for caregiver-reported diarrhoeal illness in children, using nationally representative household surveys

**DOI:** 10.1093/ije/dyz043

**Published:** 2019-03-25

**Authors:** Katie N Overbey, Kellogg J Schwab, Natalie G Exum

**Affiliations:** Department of Environmental Health and Engineering, Johns Hopkins Bloomberg School of Public Health, Johns Hopkins University, Baltimore, MD, USA

**Keywords:** Diarrhoea, period prevalence, recall, water, sanitation, hygiene, survey methodology, outcome assessment

## Abstract

**Background:**

Diarrhoeal outcomes in children are often ascertained using caregiver-reported symptoms, which are subject to a variety of biases and methodological challenges. One source of bias is the time window used for reporting diarrhoeal illness and the ability of caregivers to accurately recall episodes in children.

**Methods:**

Diarrhoea period prevalence in children under five was determined using two similarly administered, nationally representative household surveys: Performance Monitoring and Accountability 2020 (PMA2020) (1-week recall, *N* = 14 603) and Demographic and Health Surveys (DHS) (2-week recall, *N* = 66 717). Countries included in the analysis were the Democratic Republic of the Congo, Ethiopia, Ghana, Kenya and Uganda. Diarrhoea period prevalence estimates were compared and water, sanitation and hygiene risk factors were analysed.

**Results:**

Childhood diarrhoea prevalence using 1-week recall (PMA2020) pooled across countries was 21.4% [95% confidence interval (CI): 19.9%, 22.9%] versus 16.0% using 2-week recall (DHS) (95% CI: 15.4%, 16.5%). In stratified analyses for all five countries, the number of diarrhoea cases detected was consistently higher using 1-week recall versus 2-week recall. The key risk factors identified in the PMA2020 data that were not associated with diarrhoeal episodes or were attenuated in the DHS data included: the main sanitation classifications for households, disposal method used for child faeces, number of household members and wealth quintiles.

**Conclusions:**

For nationally representative household surveys assessing childhood diarrhoea period prevalence, a 2-week recall period may underestimate diarrhoea prevalence compared with a 1-week period. The household sanitation facility and practices remain key risk factors for diarrhoeal disease in children under five.

**Key Messages**

- Diarrhoea period prevalence measured by caregiver-reported recall may be underestimated when using a 2-week period versus a 1-week period.
- For large-scale, nationally-representative household surveys where diarrhoea is endemic, a 1-week recall period is recommended for measuring caregiver-reported diarrhoea period prevalence.
- Risk factors for the odds of diarrhoea display attenuation towards the null for a 2-week recall period compared with 1-week recall, suggesting increased measurement error for a longer reporting period.
- Using a 1-week recall period to measure diarrhoeal outcomes, sanitation remains a key risk factor for diarrhoeal disease in children.

## Introduction

Diarrhoeal disease remains a leading cause of morbidity and mortality in children under five.^1^^,^^2^ Globally, there are nearly 1.7 billion cases of childhood diarrhoeal disease every year and it is responsible for the deaths of around 525 000 children annually.^3^ Surveys that measure diarrhoeal illness and associated risk factors on a nationally representative scale are critical to understand the diarrhoea burden and potential impact of interventions.

To study the burden of diarrhoeal disease on a population over time and estimate the relationships between water, sanitation and hygiene (WASH) exposures and diarrhoeal outcomes, the cross-sectional cohort design may offer advantages to measure diarrhoeal episodes.^4^ Diarrhoeal outcomes in children are often ascertained using caregiver-reported symptoms, which are subject to a variety of biases and methodological challenges.^5^^,^^6^ One source of bias is the time window used for reporting diarrhoeal illness and the ability of caregivers to accurately recall diarrhoeal episodes.^5^^,^^7^^,^^8^ For large, nationally representative surveys that use the cross-sectional cohort design, longer symptom recall periods are typically used, to increase the number of diarrhoea cases detected and reduce the outcome variability of period prevalence over the measurement period.^9^^,^^10^ Longer recall periods are also likely to introduce bias due to measurement error. For diarrhoea morbidity in children, recall periods longer than 3 days will likely increase under-reporting of diarrhoea as the number of days to recall symptoms increases.^11–15^ A 2-week recall period for diarrhoeal episodes is a standard used in national surveys that measure diarrhoeal disease.^16–19^ A shorter recall period may increase accuracy; however, it may also require a significant increase in sample size, negatively impacting the variance of estimates and reducing statistical power.^10^^,^^12^ Previous work has suggested that a 1-week recall period may optimally balance statistical power with reduction of recall bias.^20^ Though previous studies have used a 1-week recall period for measuring diarrhoea prevalence,^21^^,^^22^ the outcome has not been analysed across large, nationally representative surveys to understand the influence of recall period on disease prevalence and its impact on the determination of diarrhoeal disease risk factors.

The objectives of this study were: first, to compare caregiver-reported diarrhoea period prevalence from two similarly administered national surveys that use 1-week and 2-week recall periods; and second, to determine how these recall periods impact the relationships between known household WASH risk factors and diarrhoeal episodes. The two datasets came from the Performance Monitoring and Accountability 2020 (PMA2020) surveys that use a 1-week recall for ascertaining caregiver-reported diarrhoea, and the Demographic and Health Surveys (DHS) that use a 2-week recall window. The underlying assumption with the use of a 2-week recall period compared with a 1-week period is that a larger number of diarrhoea cases will be detected with a longer time window.

## Methods

To allow for the assumption of comparability between nationally representative estimates, two similarly administered, publicly available surveys were used: Performance Monitoring and Accountability 2020 (PMA2020), and Demographic and Health Surveys (DHS). PMA2020 was designed to be comparable to DHS, and the majority of questions included in the PMA2020 household and female questionnaires replicate wording from DHS, including key WASH questions.^23^ Both PMA2020 and DHS use a cross-sectional design with stratified, cluster random sampling done in collaboration with national bureaus of statistics. Interviews for both surveys are conducted by female enumerators who hold at least a secondary education and are trained in the interviewing methods necessary to administer household questionnaires to female respondents age 15–49. There were five countries from sub-Saharan Africa (the Democratic Republic of the Congo, Ethiopia, Ghana, Kenya and Uganda) which had datasets with information on diarrhoea in children under 5 years old available from both surveys and were collected within 2 years of each other (Table 1). Both surveys interviewed caregivers of children under 5 years old about previous episodes of diarrhoea, as perceived by the caregiver. If unsure, in both surveys they were informed that diarrhoea means three or more runny stools per day. The surveys used different recall periods, with PMA2020 using a 1-week time window and DHS using a 2-week window.

**Table 1.** Datasets used in analysis of diarrhoea prevalence in children under 5 years of age

	PMA2020	DHS
Democratic Republic of the Congo	Round 4, 2015-16	Phase 6, 2013-14
Ethiopia	Round 4, 2016	Phase 7, 2016
Ghana	Round 3, 2014	Phase 7, 2014
Kenya	Round 4, 2015	Phase 7, 2014
Uganda	Round 4, 2016	Phase 7, 2016

### PMA2020 data

PMA2020 collects data annually on key WASH indicators as defined by the WHO/UNICEF Joint Monitoring Programme (JMP).^24^ PMA2020 employs resident female enumerators and mobile technology to collect data on a range of family planning, WASH and health issues. The sample selection, household surveys and mobile platform have been previously described in detail.^23^^,^^25^ Briefly, a multistage cluster sample was used to draw a probability sample of households. All households received a questionnaire that included questions on demographics, assets and WASH characteristics. All females aged 15–49 listed on the household roster were administered the female questionnaire which included questions on childhood diarrhoeal outcomes. Full questionnaires can be found at [https://pma2020.org/questionnaires].

Datasets were downloaded from [www.pma2020.org], and scripts for reproducibility are included in Supplementary materials (available as Supplementary data at *IJE* online). Datasets from the Democratic Republic of the Congo Round 4 (2015–16), Ethiopia Round 4 (2016), Ghana Round 3 (2014), Kenya Round 4 (2015) and Uganda Round 4 (2016) were used. All datasets were combined into one for a total of 14 603 children from 10 754 unique households.

### PMA2020 analysis

Diarrhoeal outcomes were ascertained for each child under five in the household by asking their female caregiver ‘In the past 7 days, has this child had diarrhoea?’ Households without children were removed from the analysis; ‘I don’t know’ and ‘other’ responses were coded as missing. Independent variables for analysis were pre-selected based on risk factors identified in previous literature^26–29^ and availability in the PMA2020 datasets. This resulted in the following set of independent variables: country, urban/rural, number of household members, number of children under five in household, household wealth quintile, caregiver’s highest education level, child’s age, main drinking water source classification, drinking water reliability, main sanitation facility classification, method used for disposal of children’s faeces and presence of handwashing stations.

Caregiver education was standardized by age and grade levels across all countries. The following education categories were applied: never attended, primary, secondary/middle, college/university/higher education, post-primary/vocational. Improved and unimproved household drinking water sources and sanitation facilities were classified based on JMP definitions.^30^ Sanitation facilities were classified as improved and not shared, improved and shared, unimproved or open defaecation. Water reliability was reported for the household’s main drinking water source and was classified as always available, intermittent predictable and intermittent unpredictable. Each child faeces management practice was treated as a binary variable and included the following options: burn, bury, child uses latrine, faeces disposed in latrine, garbage, leave child’s faeces, use for manure and dispose of faeces in waste water. Handwashing location was self-reported and was either none, designated place or use of a movable container. Child’s age was analysed in months and the final model included splines at 6, 12, 18 and 24 months of age.^27^^,^^29^^,^^31^

We conducted a risk factor analysis to examine the associations between household WASH characteristics and diarrhoea in children under five. All statistical analyses were conducted using Stata version 13.1 (StataCorp, 2013). Data were weighted using probability sample weights to account for the random, multicluster sampling strategy. The effects of household level clustering were examined using a mixed effects model with random intercepts for enumeration area and household. Estimates were similar to those when only controlling for clustering at enumeration area level, so the latter method was used.

Bivariate relationships between all independent variables and the diarrhoeal outcome were analysed. Unweighted forward and backward stepwise selection was used in combination with content knowledge to develop a final parsimonious model for multivariate analysis. Due to the absence of appropriate model diagnostics for survey data, model diagnostics were not used to compare models for final selection. Instead, fit was evaluated using the Archer-Lemeshow test, which is a modified Hosmer-Lemeshow goodness-of-fit test, and by examining weighted deviance residuals versus fitted values.^32^ For the final selected model, the Archer-Lemeshow test was not significant and no trends were observed in the residual versus fitted values.

### DHS data

The DHS Program collects data on indicators for population, health and nutrition. Surveys used in this study are DHS Standard Surveys, which are nationally representative surveys collected approximately every 5 years in over 90 countries. Information on sample selection and survey administration has been described in detail.^33^

DHS datasets were downloaded from [https://www.dhsprogram.com/] and the scripts for reproducibility can be found in Supplementary materials (available as Supplementary data at *IJE* online). The DHS Phase 6 questionnaire was used for the Democratic Republic of the Congo (2013–14), and the DHS Phase 7 questionnaire was used for Ghana (2014), Kenya (2014), Ethiopia (2016) and Uganda (2016). A total of 66 717 children from 44 349 unique households were analysed.

Diarrhoeal outcomes were ascertained for each child under five in the household by asking their female caregiver ‘Has (child’s name) had diarrhoea in the last 2 weeks?’ Maternal education was classified as none, primary, secondary and higher/university. For all variables, if the respondent said they were not a usual resident of the household, the variable was reported as missing. The DHS dataset included child’s gender and time to water source, which were not reported in PMA2020. DHS did not report water reliability or presence of a handwashing location. Probability sampling weights were applied and period prevalence estimates for diarrhoea were compared with the PMA2020 data. Bivariate relationships between all independent variables and the diarrhoeal outcome were also analysed.

### Comparison of PMA2020 and DHS data

A multivariate logistic regression model based on the final PMA2020 model was applied to DHS data and resulting adjusted odds ratios were compared. This model included all variables from the final PMA2020 model, except those not found in the DHS dataset (presence of handwashing location). Time required to collect water was included in the DHS model as a proxy for the PMA2020 water reliability variable. This was reported in minutes, and if water was on premises this was ‘0’.

## Results

### Trends of diarrhoea period prevalence

**Figure 1.**
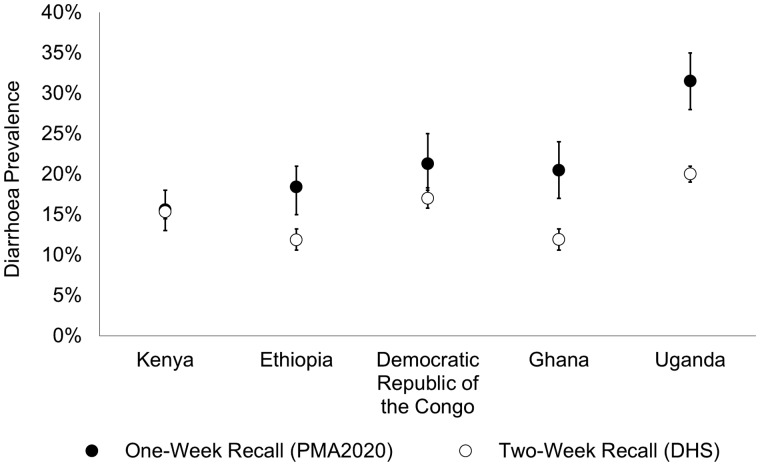
Comparison of weighted estimates of diarrhoea prevalence in children under five by country between PMA2020 data (1-week recall period) and DHS data (2-week recall period), error bars indicate 95% confidence intervals.

**Figure 2.**
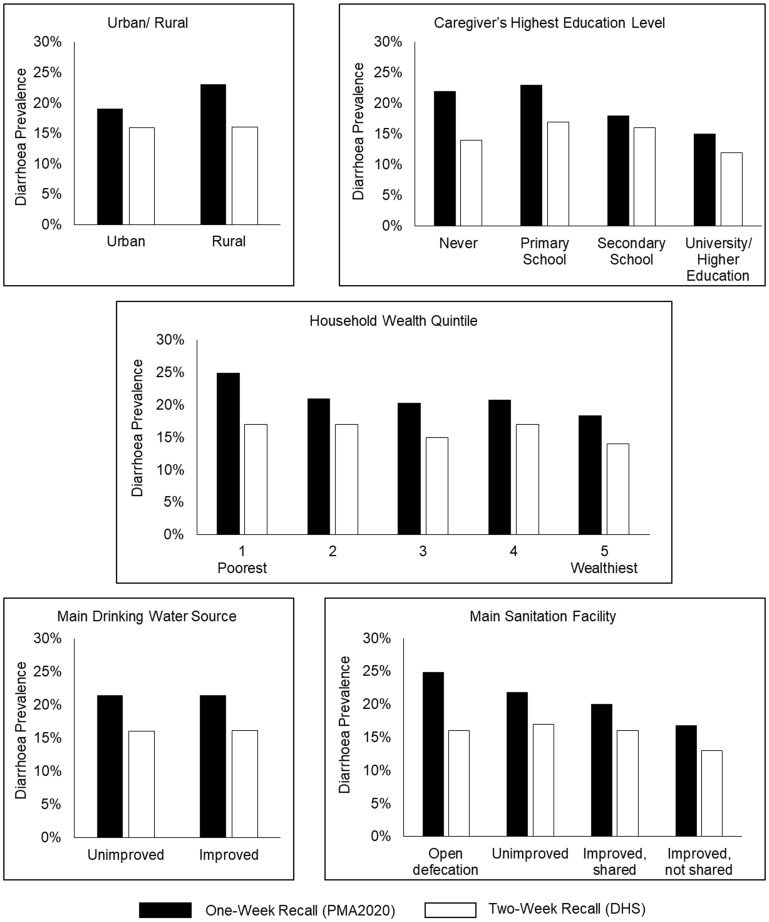
Comparison of diarrhoea prevalence in children under five using PMA2020 data (1-week recall period) and DHS data (2-week recall period), across selected categorical variables.

Weighted period prevalence of diarrhoea in children under 5 years old across all countries using PMA2020 data (1-week recall period) was 21.4% (95% CI: 19.9%, 22.9%) and 16.0% using DHS data (2-week recall period) (95% CI: 15.4%, 16.5%). Demographic and household WASH characteristics are shown in Table 2 (unweighted frequencies). Diarrhoea period prevalence in each country was consistently higher in the PMA2020 data compared with DHS (Figure 1). For both datasets, the weighted diarrhoea period prevalence was highest in Uganda and lowest in Kenya (Table 3). The greatest difference in period prevalence between surveys was in Uganda, where PMA2020 was 11.5% higher than DHS and the smallest difference was in Kenya, where PMA2020 was 0.2% higher (Table 3). Childhood diarrhoea period prevalence estimates were stratified by demographic and WASH characteristics in both datasets (Figure 2). For all measured variables, diarrhoea period prevalence was consistently higher using PMA2020 data.

**Table 2.** Sample breakdown of selected demographic and household water, sanitation, and hygiene characteristics for children included in analysis

Characteristic	PMA2020 *N* = 14 603	DHS *N *= 66 717
Country		
Democratic Republic of the Congo	16.8%	25.7%
Ethiopia	31.6%	15.9%
Ghana	13.0%	8.2%
Kenya	17.8%	28.4%
Uganda	20.7%	21.7%
		
Development type		
Urban	37.2%	28.1%
Rural	62.8%	71.9%
Average number of members in household (SE)	6.35 (0.07)	6.18 (0.03)
Average number of children aged <5 years in household (SE)	1.62 (0.02)	1.92 (0.01)
Wealth quintile	*n* = 14 578^a^	
Poorest	24.6%	23.0%
Second Poorest	22.1%	21.4%
Middle	20.3%	19.5%
Second Wealthiest	17.1%	18.0%
Wealthiest	15.9%	18.2%
Caregiver’s education level	*n* = 14 599^a^	
None	25.2%	23.3%
Primary school	42.4%	46.3%
Secondary school	21.7%	25.4%
Vocational training	6.6%	–
University/higher education	4.2%	5.0%
Child's gender		
Female	–	49.5%
Male	–	50.5%
Child's age	*n* = 14 362^a^	
Average age of child in months (SE)	28.8 (0.20)	29.0 (0.08)
Main drinking water source classification	*n* = 14 597^a^	*n* = 65 035^a^
Unimproved	27.4%	35.4%
Improved	72.6%	64.6%
Time to get water		*n* = 65 039^a^
Time in minutes (SE)	–	31.8 (0.66)
Reliability	*n* = 14 596^a^	
Always	71.5%	–
Intermittent predictable	16.9%	–
Intermittent unpredictable	11.6%	–
Main sanitation classification		*n* = 65 209^a^
Open defecation	17.8%	17.8%
Unimproved	51.3%	44.3%
Improved, shared	18.4%	21.9%
Improved, not shared	12.5%	16.1%
Child faeces disposal		*n* = 45 719^a^
Burn	0.3%	–
Bury	9.2%	4.1%
Child uses latrine	20.8%	6.0%
Disposed of in latrine	57.1%	56.8%
Garbage	17.2%	18.3%
Leave	8.5%	8.5%
Manure	0.5%	–
Waste water	13.2%	6.2%
Handwashing	*n* = 14 250^a^	
None	52.1%	–
Designated place	23.3%	–
Container	24.7%	–

SE, standard error of the mean.

a Sample size reduced due to missing values.

**Table 3.** Weighted estimates of diarrhoea period prevalence in children under five by country, comparison of surveys using a 1-week recall period (PMA2020) and a 2-week recall period (DHS)

	PMA2020			DHS			
Country	Period prevalence (%)	95% CI	*N*	Period prevalence (%)	95% CI	*N*	Percent difference^a^
Democratic Republic of the Congo	21.3	(18.0, 24.6)	2591	17.0	(15.8, 18.3)	16 994	4.3
Ethiopia	18.4	(15.4, 21.4)	4147	11.9	(10.6, 13.2)	9916	6.5
Ghana	20.5	(17.1, 23.9)	2237	11.9	(10.6, 13.2)	5539	8.6
Kenya	15.6	(12.9, 18.4)	2655	15.4	(14.5, 16.2)	19 889	0.2
Uganda	31.5	(28.1, 35.0)	3059	20.0	(19.0, 21.0)	14 379	11.5

a Difference in weighted period prevalence estimates between PMA2020 and DHS data.

### Diarrhoea risk factors identified in PMA2020 data

Children in homes with improved sanitation had lower odds of diarrhoea than children in homes with no sanitation facilities, in both unadjusted [odds ratio (OR): 0.61; 95% CI: 0.47, 0.80] and adjusted odds ratio (AOR) analyses (AOR: 0.68; 95% CI: 0.49, 0.94) measured by PMA2020 (Table 4). There was no difference in the odds for diarrhoeal illness between households with improved versus unimproved drinking water sources (OR: 1.00; 95% CI: 0.82, 1.20) (Table 4). Lower odds of diarrhoea were associated with the presence of a designated handwashing station, compared with households without a handwashing location, in both unadjusted (OR: 0.64; 95% CI: 0.52, 0.79) and adjusted models (AOR: 0.80; 95% CI: 0.63, 1.02) (Table 4).

**Table 4.** Results of multivariate logistic regression analysis of diarrhoea among children younger than 5 years with selected water, sanitation and hygiene risk factors using 1-week (PMA2020) and 2-week (DHS) diarrhoea recall period data

	1-week recall period data (PMA2020)				2-week recall period data (DHS)			
	Unadjusted odds ratio (95% CI)	*P*-value	Adjusted odds ratio^a^ (95% CI)	*P*-value	Unadjusted odds ratio (95% CI)	*P-*value	Adjusted odds ratio^a^ (95% CI)	*P*-value
Country								
Democratic Republic of Congo	REF		REF		REF		REF	
Ethiopia	0.84 (0.63, 1.11)	0.21	0.76 (0.54, 1.05)	0.097	0.66 (0.56, 0.77)	<0.001	0.56 (0.46, 0.68)	<0.001
Ghana	0.95 (0.72, 1.27)	0.75	0.88 (0.65, 1.19)	0.41	0.66 (0.56, 0.77)	<0.001	0.64 (0.54, 0.76)	<0.001
Kenya	0.69 (0.51, 0.91)	0.01	0.66 (0.49, 0.89)	0.006	0.89 (0.79, 0.99)	0.028	0.9 (0.79, 1.03)	0.14
Uganda	1.71 (1.32, 2.20)	<0.001	1.42 (1.06, 1.91)	0.019	1.22 (1.10, 1.36)	<0.001	1.28 (1.13, 1.45)	<0.001
Development type								
Urban	REF		–	–	REF		–	–
Rural	1.24 (1.04, 1.48)	0.015	–	–	1.05 (0.97, 1.14)	0.23	–	–
Number of household members	1.04 (1.02, 1.06)	<0.001	1.03 (1.01, 1.05)	0.0041	1 (0.98, 1.01)	0.53	0.99 (0.98, 1.01)	0.38
Number of children <5 years old in household	1.04 (0.96, 1.12)	0.36	–	–	1.03 (1.00, 1.06)	0.076	–	–
Household wealth (quintile)								
1 Poorest	REF		REF		REF		REF	
2	0.80 (0.68, 0.95)	0.01	0.83 (0.70, 0.98)	0.03	0.98 (0.90, 1.06)	0.57	0.97 (0.87, 1.08)	0.6
3	0.77 (0.64, 0.93)	0.006	0.81 (0.66, 0.98)	0.032	0.9 (0.82, 0.98)	0.02	0.95 (0.84, 1.08)	0.44
4	0.79 (0.64, 0.97)	0.026	0.87 (0.70, 1.09)	0.22	0.98 (0.87, 1.09)	0.66	1.07 (0.93, 1.24)	0.34
5 Wealthiest	0.68 (0.55, 0.85)	<0.001	0.85 (0.66, 1.08)	0.18	0.77 (0.70, 0.86)	<0.001	0.88 (0.75, 1.02)	0.091
Caregiver’s highest education level								
None	REF		REF		REF		REF	
Primary school	1.1 (0.93, 1.32)	0.27	0.97 (0.81, 1.17)	0.77	1.34 (1.22, 1.47)	<0.001	1.11 (0.99, 1.25)	0.08
Secondary school	0.77 (0.63, 0.95)	0.015	0.77 (0.62, 0.95)	0.014	1.24 (1.12, 1.37)	<0.001	1.14 (0.99, 1.30)	0.064
Vocational training	1.17 (0.90, 1.52)	0.24	0.92 (0.71, 1.18)	0.5	–	–	–	–
University/higher education	0.65 (0.46, 0.91)	0.013	0.82 (0.58, 1.15)	0.25	0.85 (0.71, 1.02)	0.073	0.95 (0.76, 1.19)	0.65
Child's gender^b^								
Female	–	–	–	–	REF		–	–
Male	–	–	–	–	1.15 (1.09, 1.22)	<0.001	–	–
Child's age (months)								
0-5	1.16 (1.08, 1.24)	<0.001	1.14 (1.07, 1.22)	<0.001	1.46 (1.40, 1.52)	<0.001	1.46 (1.39, 1.53)	<0.001
6-11	1.04 (0.99, 1.08)	0.15	1.04 (0.99, 1.09)	0.11	1.02 (0.99, 1.04)	0.13	1.03 (1.00, 1.06)	0.023
12-17	0.98 (0.93, 1.02)	0.31	0.97 (0.93, 1.02)	0.29	0.95 (0.93, 0.98)	<0.001	0.95 (0.92, 0.97)	<0.001
18-23	0.95 (0.91, 0.99)	0.012	0.95 (0.91, 0.99)	0.014	0.95 (0.93, 0.97)	<0.001	0.95 (0.93, 0.97)	<0.001
>=24	0.98 (0.97, 0.98)	<0.001	0.98 (0.97, 0.98)	<0.001	0.96 (0.96, 0.96)	<0.001	0.96 (0.95, 0.96)	<0.001
Main drinking water source classification								
Unimproved	REF		–	–	REF		–	–
Improved	1.00 (0.82, 1.20)	0.96	–	–	1.01 (0.93, 1.09)	0.88	–	–
Water reliability^c^								
Always	REF		REF		–	–	–	–
Intermittent predictable	1.12 (0.91, 1.37)	0.27	1.15 (0.95, 1.40)	0.16	–	–	–	–
Intermittent unpredictable	1.04 (0.82, 1.32)	0.72	1.15 (0.93, 1.43)	0.20	–	–	–	–
Time to get water (min)^b^	–	–	–	–	1 (1.00, 1.00)	<0.001	1 (1.00, 1.00)	0.05
Main sanitation classification								
Open defecation	REF		REF		REF		REF	
Unimproved	0.85 (0.68, 1.05)	0.13	0.83 (0.64, 1.09)	0.18	1.06 (0.96, 1.16)	0.26	0.92 (0.81, 1.04)	0.2
Improved, shared	0.76 (0.59, 0.98)	0.03	0.84 (0.63, 1.14)	0.27	1.04 (0.93, 1.15)	0.52	0.96 (0.83, 1.12)	0.62
Improved, not shared	0.61 (0.47, 0.80)	<0.001	0.68 (0.49, 0.94)	0.02	0.78 (0.69, 0.88)	<0.001	0.7 (0.60, 0.83)	<0.001
Child faeces disposal^d^								
Burn	1.36 (0.65, 2.85)	0.42	–	–	–	–	–	–
Bury	1.36 (1.05, 1.75)	0.019	–	–	1.25 (1.07, 1.44)	0.0037	–	–
Child uses latrine	0.80 (0.67, 0.95)	0.013	0.82 (0.68, 0.99)	0.04	0.57 (0.48, 0.69)	<0.001	0.89 (0.74, 1.07)	0.21
Disposed of in latrine	0.99 (0.84, 1.16)	0.88	–	–	1.17 (1.08, 1.26)	<0.001	–	–
Garbage	0.90 (0.74, 1.08)	0.25	0.89 (0.72, 1.09)	0.25	0.85 (0.77, 0.95)	0.0025	0.94 (0.83, 1.06)	0.29
Leave	1.15 (0.87, 1.52)	0.32	–	–	0.99 (0.86, 1.14)	0.88	–	–
Manure	0.68 (0.23, 2.00)	0.49	–	–	–	–	–	–
Waste water	1.02 (0.83, 1.25)	0.84	0.93 (0.76, 1.15)	0.5	1.07 (0.93, 1.23)	0.34	1.12 (0.97, 1.30)	0.12
Handwashing^c^								
None	REF		REF		–	–	–	–
Designated place	0.64 (0.52, 0.79)	<0.001	0.80 (0.63, 1.02)	0.076	–	–	–	–
Container	0.86 (0.72, 1.04)	0.12	0.91 (0.76, 1.09)	0.29	–	–	–	–

a Adjusted for country, number of household members, household wealth quintile, caregiver’s highest education level, child’s age with splines at 6, 12, 18 and 24 months, water reliability, main sanitation facility classification, child faeces disposal practices: child uses latrine, disposed in garbage, disposed in waste water, and presence of handwashing location.

b Variables in the 2-week (DHS) diarrhoea recall dataset only.

c Variables in the 1-week (PMA2020) diarrhoea recall dataset only.

d Child faeces management practices were treated as binary variables, where households reporting each practice were compared with all households that did not report that practice.

In households where children used the latrine for faeces disposal, the odds of diarrhoea were lower than in households where children did not use the latrine (AOR: 0.82; 95% CI: 0.68, 0.99). In bivariate analyses, burying child faeces increased odds of diarrhoea by 36% (95% CI: 1.05, 1.75); however this variable was not included in the final model due to limited sample size.

### Comparison of risk factors for diarrhoea between PMA2020 and DHS

In unadjusted risk factor analyses for both PMA2020 and DHS surveys, the following WASH factors were associated with diarrhoea in children under five: main sanitation facility classification, child faeces disposal by burying, disposing in a latrine or in garbage, having children use the latrine and presence of a handwashing station in a designated place (Table 4).

**Figure 3.**
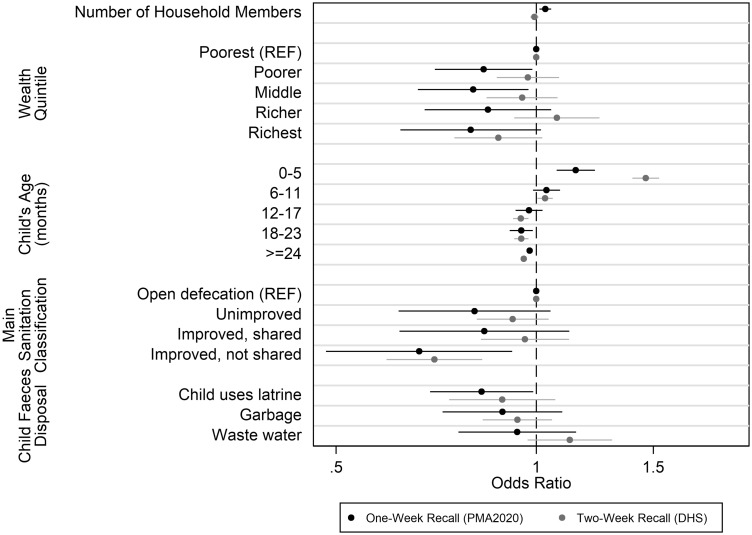
Comparison of adjusted odds ratios for select independent variables from multivariate logistic regression for diarrhoea among children younger than 5 years between PMA2020 (1-week recall) and DHS (2-week recall), error bars indicate 95% confidence intervals. Full model also adjusted for country and education (not displayed). Additionally, PMA2020 model includes water reliability, whereas DHS model includes time to collect water as a proxy for water reliability. Presence of a handwashing location was also not available in the DHS data.

PMA2020 and DHS datasets were compared using the same multivariate logistic regression model based on the final PMA2020 model (Figure 3). This model included country of residence, number of household members, household wealth quintile, caregiver’s highest education level, child’s age, time to water (DHS)/drinking water reliability (PMA2020), main sanitation facility classification and child faeces management practices (child uses latrine, disposed of in garbage, disposed of in waste water). The adjusted odds ratios for diarrhoea using DHS data were attenuated towards the null for the number of household members, household wealth quintile, sanitation classification and child faeces disposal risk factors.

## Discussion

A consistent underestimation of diarrhoea period prevalence was found for nationally representative surveys when comparing the DHS 2-week recall period with the PMA2020 1-week recall period. This finding is contrary to the assumption that a longer recall window will increase the number of cases detected and result in a larger diarrhoea period prevalence as measured by DHS. Previous literature suggests this finding may be due to symptom recall bias from certain aspects of human memory.^13^ First, it is possible that ‘telescoping’ of memory occurred such that diarrhoeal symptoms were remembered as occurring more recently than they actually did.^34^^,^^35^ In this case, events that were beyond the 7-day window might have been recalled as having occurred during the 1-week time window used in PMA2020. Second, it is possible that it was more difficult for caregivers to remember diarrhoeal episodes when given a 2-week time period as opposed to a 7-day time period. Caregivers may have had an easier time remembering diarrhoeal symptoms when recollecting personal events in the past 7 days, which is a standard unit of time in many cultures (1 week), and might have led to greater accuracy.^34^ Different reporting tendencies have been found in countries with high levels of diarrhoea, where less severe cases are more often reported.^12^ The increased accuracy of a 7-day time period, along with less severe cases being reported more often where diarrhoea is highly prevalent, may explain why Uganda, with the highest diarrhoea period prevalence, had the greatest difference in prevalence between PMA2020 and DHS data.

A comparison of the multivariate logistic regression models showed that adjusted odds ratios were attenuated towards the null for a number of risk factors in DHS data when compared with PMA2020 data (Figure 3). The key risk factors identified in the PMA2020 data, which were not associated with diarrhoeal episodes or were attenuated in the DHS data, included: the main sanitation classifications for households, disposal method used for child faeces, number of household members and wealth quintiles. These differences in key risk factors may be due to measurement error from the recall period. Previous research in the context of survey measurement has found that event recall is less accurate over time.^8^^,^^36^^,^^37^ Findings from Stull *et al.* also determined that an incorrect recall period introduces measurement error that may reduce the chances of detecting a treatment effect.^8^

In the PMA2020 risk factor analysis, children in homes with improved sanitation had lower odds of diarrhoea than children in homes with no sanitation facilities in both unadjusted and adjusted analyses. This is in agreement with previous studies that identified household sanitation practices as important drivers of diarrhoeal outcomes.^38^^,^^39^ Also in the PMA2020 analysis, drinking water source was not associated with diarrhoeal illness. This is also consistent with previous work that shows that sanitation has a larger role in reduction of diarrhoeal illness than drinking water source.^40^ Günther and Fink used 172 DHS datasets from 70 countries, and found that the odds of children having diarrhoea were most significantly reduced by sanitation infrastructure rather than by basic water supply.^41^ Cairncross *et al.* drew on systematic reviews and proposed diarrhoea risk reductions of 36% for improved excreta disposal and 17% for improved water supply.^40^ In the PMA2020 analysis, a household that used a latrine to dispose of a child’s faeces reduced the odds of diarrhoea for children in that household, even when controlling for type of household sanitation and child’s age. This in line with previous evidence that disposing of child faeces in a latrine reduces the odds of childhood diarrhoea.^27^ Presence of a designated handwashing location was found to reduce the odds of diarrhoea in children under five in PMA2020 data (Table 4), which is supported by previous studies. Kamm *et al*. found that the presence of soap in a home was associated with a reduction in diarrhoeal illness,^42^ and Wilson and Chandler found that self-reported soap use by mothers was associated with decreased rates of diarrhoea.^43^ These findings from the risk factor analysis demonstrate an attenuation towards the null for the main WASH risk factors associated with diarrhoea, when using the 2-week recall period in DHS data compared with 1-week recall period in PMA2020 data. This has important implications for future nationally representative surveys in developing countries that want to track progress in driving down diarrhoeal disease, where WASH interventions play a necessary role to accomplish this goal. To improve measurement of diarrhoea period prevalence and the associated risk factors, a 1-week recall period may be preferred over a 2-week period.

To our knowledge, this study is the first to compare nationally representative surveys that use different recall time windows (1-week versus 2-week) for diarrhoea period prevalence in children under 5 years old. The large sample sizes in both PMA2020 and DHS datasets increase confidence in our findings that a 2-week recall period for diarrhoeal episodes may underestimate diarrhoea period prevalence in nationally representative estimates. Comparability was enabled by the high standardization in survey methodology between PMA2020 and DHS, including the cross-sectional design with stratified, cluster random sampling, probability weighting, administration of household questionnaires and similar formulation of key WASH questions. There were some temporal variations between the two datasets, where PMA2020 surveys were collected more recently than DHS (Table 1). Given the global downward trend in diarrhoeal disease,^44^^,^^45^ more recent data collection in a country is expected to result in slightly lower diarrhoea prevalence. Therefore in the Democratic Republic of the Congo and in Kenya, where PMA2020 was collected 2 years and 1 year, respectively, after DHS data were collected, our finding of higher diarrhoea prevalence in PMA2020 data when compared with DHS data is even more unexpected. A second limitation related to the temporal nature of the data is that the fieldwork of DHS surveys goes on for several months, up to a year, whereas many PMA2020 surveys are completed within 2 months. As diarrhoea shows a seasonal pattern in many countries, it would have been preferable to compare estimates from the surveys that matched seasons; however, such data were not available from DHS surveys. Despite the high standardization between the two surveys, to determine the optimal recall period for caregiver-reported diarrhoea prevalence ideally the recall time periods compared would be used in the same survey. Other limitations from this study include the missing values for the child faeces disposal variable that reduced sample sizes of the datasets for the risk factor analyses. Symptom severity is also known to affect diarrhoea recall,^15^ but was not collected in DHS or PMA2020 surveys. DHS does collect information on care-seeking behaviours, which could be used as a proxy for symptom severity, though PMA2020 does not collect this information. The impact of symptom severity on diarrhoea recall is important for future work to determine the optimal recall period in caregiver-reported diarrhoea. Last, these data do not discern if children had multiple episodes of diarrhoea during the recall period, and this may be another reason for underestimation of diarrhoea period prevalence.

In conclusion, we examined diarrhoea period prevalence in children under 5 years old using two nationally representative datasets pooled from low- and middle-income countries, which use 2-week and 1-week recall periods for caregiver-reported diarrhoea. Data collected using a 2-week recall period from DHS consistently underestimated diarrhoea prevalence when compared with a 1-week recall period from PMA2020. This finding indicates that choice of recall period for ascertaining caregiver-reported diarrhoea has a potentially significant impact on prevalence measures. For countries where national surveys are administered to measure reductions in the burden of diarrhoeal illness in children, a 1-week recall period may more accurately determine disease period prevalence and risk factors, for points of intervention.

## Funding

This work was supported by the Osprey Foundation of Maryland and the National Institutes of Health [T32 ES007141]. PMA2020 is funded through a grant from the Bill & Melinda Gates Foundation [grant number 114805].

## Supplementary Material

dyz043_Supplementary_Data
